# Health status outcomes after spontaneous coronary artery dissection and comparison with other acute myocardial infarction: The VIRGO experience

**DOI:** 10.1371/journal.pone.0265624

**Published:** 2022-03-23

**Authors:** Karthik Murugiah, Lian Chen, Rachel P. Dreyer, Georgios Bouras, Basmah Safdar, Rohan Khera, Yuan Lu, Erica S. Spatz, Vivian G. Ng, Aakriti Gupta, Héctor Bueno, Marysia S. Tweet, John A. Spertus, Sharonne N. Hayes, Alexandra Lansky, Harlan M. Krumholz

**Affiliations:** 1 Section of Cardiovascular Medicine, Department of Internal Medicine, Yale School of Medicine, New Haven, CT, United States of America; 2 Center for Outcomes Research and Evaluation, Yale-New Haven Hospital, New Haven, CT, United States of America; 3 Department of Emergency Medicine, Yale School of Medicine, New Haven, CT, United States of America; 4 417 NIMTS Veterans’ Fund Hospital of Athens, Athens, Greece; 5 Division of Cardiovascular Medicine, Columbia University Irving Medical Center, New York, NY, United States of America; 6 Cedars-Sinai Medical Center, Smidt Heart Institute, Los Angeles, CA, United States of America; 7 Centro Nacional de Investigaciones Cardiovasculares (CNIC), Madrid, Spain; 8 Instituto de Investigacion i+12 and Cardiology Department, Hospital Universitario 12 de Octubre, Madrid, Spain; 9 Facultad de Medicina, Universidad Complutense de Madrid, Madrid, Spain; 10 Department of Cardiovascular Diseases, Mayo Clinic College of Medicine and Science, Rochester, MN, United States of America; 11 Saint Luke’s Mid America Heart Institute, Kansas City, MO, United States of America; 12 University of Missouri-Kansas City, Kansas City, MO, United States of America; 13 Yale School of Public Health, New Haven, CT, United States of America; Rutgers Robert Wood Johnson Medical School, UNITED STATES

## Abstract

**Background:**

Data on health status outcomes after spontaneous coronary artery dissection (SCAD) are limited.

**Methods and findings:**

Using the Variation in Recovery: Role of Gender on Outcomes of Young AMI Patients (VIRGO) study we compared patients with SCAD and other acute myocardial infarction (AMI) at presentation (baseline), 1-month, and-12 months using standardized health status instruments. Among 3572 AMI patients ≤ 55 years, 67 had SCAD. SCAD patients were younger (median age (IQR) 45 (40.5–51) years vs. 48 (44–52) in other AMI, p = 0.003), more often female (92.5% vs. 66.6%), have college education (73.1% vs. 51.7%) and household income >$100,000 (43.3% vs. 17.7% (All p<0.001). SCAD patients at baseline had higher mean ± SD Short Form-12 [SF-12] physical component scores [PCS] (48.7±10.2 vs. 43.8±12.1, p<0.001) and mental component scores [MCS] (49.6±12.4 vs. 45.4±12.5, p = 0.008), and at 12-months [PCS (50.1±9.0 vs. 44.3±12.3, p<0.001) and MCS (53±10.1 vs 50.2±11.0, p = 0.045)]. The Euro-Quality of Life Scale [EQ-5D] VAS and EQ-5D index scores were similar at baseline, but higher at 12-months for SCAD (EQ-5D VAS: 82.2±10.2 vs. 72.3±21.0, p<0.001; EQ-5D index scores; 90.2±15.3 vs. 83.7±19.8, p = 0.012). SCAD patients had better baseline Seattle Angina Questionnaire [SAQ] physical limitation (88.8±20.1 vs. 81.2±25.4, p = 0.017). At 12-months SCAD patients had better physical limitation (98.0±8.5 vs. 91.4±18.8, p = 0.007), angina frequency (96.4±8.8 vs. 91.3±16.8, p = 0.018) and quality of life scores (80.7±14.7 vs 72.2±23.2, p = 0.005). Magnitude of change in health status from baseline to 12-months was not statistically different between the groups. After adjustment for time and comorbidities there remained no difference in most health status outcomes.

**Conclusions:**

SCAD patients fare marginally better than other AMI patients on most health status instruments and have similar 12-month health status recovery. Better pre-event health status suggests a need to modify exercise prescriptions and cardiac rehabilitation protocols to better assist this physically active population to recover.

## Introduction

Spontaneous coronary artery dissection (SCAD) is an infrequent but increasingly recognized cause of acute coronary syndrome. SCAD most commonly affects younger women with some studies reporting prevalence of up to 35% among women under 50 years of age with acute myocardial infarction (AMI) [1, 2]. Mortality outcomes for this condition are low with the largest series reporting a 30-day mortality of 0.1% [3] and 1-year mortality of 1.1% [4], however, similar to patients with AMI, patients with SCAD remain at elevated risk for major adverse cardiovascular events (MACE) [3–5].

Our knowledge regarding outcomes among SCAD patients, however, is primarily limited to mortality and MACE which are insufficient to fully understand patients’ experiences after AMI. Health status outcomes after SCAD, such as symptoms, functional status, and quality of life, are important to patients but less well documented [6–8]. Another limitation of prior studies on SCAD is that they have been often restricted to either single-center case series or lack a comparable AMI group to provide context for the reported outcomes [2–8].

Accordingly, we used data from the Variation in Recovery: Role of Gender on Outcomes of Young AMI Patients (VIRGO) study, which is a large multi-center prospective cohort of young patients presenting with AMI. Given its target population of patients, multicenter nature, and rich collection of patient-reported outcomes, VIRGO is uniquely positioned for an in-depth characterization of health status outcomes in SCAD and allowing for a contrast with a comparable other AMI population. We compared mortality, rehospitalization and health status between SCAD and other AMI patients using standardized instruments for up to a year following the index AMI event.

## Materials and methods

### Participants and study design

The VIRGO study prospectively enrolled 3572 patients aged between 18–55 years of age hospitalized with AMI. The study used a 2:1 female:male enrollment ratio from 103 US, 24 Spanish and 3 Australian hospitals between August 2008, and January 2012 (VIRGO US grant, 5 R01 HL081153-05; VIRGO Spanish grant, 081614).

The VIRGO study has been previously described [9]. In brief, AMI was confirmed by at least 1 cardiac biomarker above the 99th percentile of the upper reference limit within 24 hours of admission along with at least 1 of the following: symptoms of ischemia, ECG changes indicative of new ischemia (new ST-T changes, new or presumably new left bundle-branch block, or the development of pathological Q waves). Patients must have presented directly to the enrolling site or must have been transferred within the first 24 hours of presentation. Patients who were incarcerated, did not speak English or Spanish, were unable to provide informed consent or to be contacted for follow-up, developed elevated cardiac markers because of elective coronary revascularization, or had an AMI as the result of physical trauma were excluded. Institutional Review Board approval was obtained at each participating institution, and patients provided written informed consent.

### SCAD identification

We identified cases of SCAD by converting the scanned medical charts for the VIRGO cohort into a Optical Character Recognition (OCR) format and searching for the phrases ‘coronary artery dissection’, ‘coronary dissection’, ‘spontaneous dissection’, and ‘SCAD’ (complete medical chart available for 3194 of 3572 patients) using a Python based script. This identified 86 charts. The cardiac catheterization reports of these 86 cases were then carefully screened by a cardiologist (K.M). Cases with iatrogenic dissection were discarded, as well as cases in which the description of the cardiac catheterization findings were inconsistent with SCAD. An example of the latter is when one of these queried terms appeared in other parts of the chart (such as the history or differential diagnosis), but the cardiac catheterization report did not mention the diagnosis as SCAD or describe an angiographic appearance/finding consistent with SCAD. This review resulted in a selected cohort of 67 patients. The clinical documentation for this cohort was then independently reviewed by a second physician (B.S), and all cases were adjudicated to have had a SCAD event.

Angiograms were available for 45 out of the 67 SCAD patients. These available angiograms were reviewed by an independent angiographic core laboratory (G.B, A.L) and 45/45 cases were confirmed to be SCAD, and evaluated in conjunction with the clinical and procedural documentation. We associated features such as visualization of dissection planes, dual lumen with delayed contrast clearing, long tubular stenoses unresponsive to vasodilators (as documented in cardiac catheterization report), or pathognomic intravascular imaging findings if performed as supportive evidence for SCAD. Given that all patients with available angiograms in the cohort were confirmed to have SCAD on angiographic review, we determined that our SCAD cohort of 67 patients identified by clinical documentation were likely SCAD. However, as elaborated in the limitations an angiogram review of the entire VIRGO cohort was not performed and many SCAD cases may have been missed.

### Data collection

As a part of VIRGO we collected patients’ sociodemographic characteristics, cardiac risk factors, disease presentation characteristics, severity, management, discharge medications from medical chart abstraction and standardized in-person interviews administered by trained personnel during the index AMI admission **(Table 1)**. In-hospital, 1-month and 12-month mortality, a well as 1-year all-cause re-hospitalizations were reported.

**Table 1:** Baseline characteristics for patients with SCAD and other AMI.

Characteristics (N, %)	Total Population (N = 3572)	SCAD (N = 67)	Other AMI (N = 3505)	P-Value
Socio-Demographics				
Age, in years (Mean±SD)	47.0±6.2	44.5±7.1	47.1±6.2	0.005
Age, in years (Median (IQR))	48 (44, 52)	45 (40.5, 51)	48 (44, 52)	0.003
**Gender, %**				
Female	2397 (67.1)	62 (92.5)	2335 (66.6)	<0.001
**Race, %**				
White	2800 (78.5)	53 (79.1)	2747 (78.4)	0.057
Black	554 (15.5)	6 (8.9)	548 (15.6)	
Other	218 (6.1)	8 (11.9)	210 (6.0)	
Hispanic*	269 (7.5)	8 (11.9)	261 (7.4)	0.167
**Marital Status, %**				<0.001
With partner	2069 (57.9)	46 (68.7)	2023 (57.7)	
Without partner	1464 (41.0)	20 (29.8)	1444(41.2)	
**Education Status, %**				
Less than high school	185 (5.2)	4 (6.0)	181 (5.2)	0.001
High school graduate	1459 (41.6)	13 (19.4)	1446 (41.3)	
More than high school	1860 (53.0)	49 (73.1)	1811 (51.7)	
**Employment Status, %**				
Working full time	1822 (51.3)	48 (71.6)	1774 (50.6)	0.002
Working part time	382 (10.7)	7 (10.4)	375 (10.7)	
Not working	1347 (37.9)	12 (17.9)	1335 (38.1)	
Has health insurance, %	2870 (80.8)	61 (91.0)	2809 (80.1)	<0.001
**Household Income**				
<10,000	603 (16.9)	1 (1.5)	602 (17.2)	<0.001
10,000–50,000	1540 (43.1)	24 (35.8	1516 (43.3)	
50,000–100,000	777 (21.8)	13 (19.4)	764 (21.8)	
>100,000	649 (18.2)	29 (43.3)	620 (17.7)	
**Cardiac Risk Factors, %**				
Hypertension	2260 (63.2)	25 (37.3)	2235 (63.8)	<0.001
Diabetes	1246 (34.9)	5 (7.5)	1241 (35.4)	<0.001
Dyslipidemia	2366 (66.2)	40 (59.7)	2326 (66.4)	0.254
Smoking within last 30 days	2133 (59.7)	14 (20.9)	2119 (60.5)	<0.001
BMI > = 30kg/m^2^	1745 (48.9)	18 (26.9)	1727 (49.3)	<0.001
Family history of CAD	2553 (71.5)	35 (52.2)	2518 (71.8)	<0.001
**Cardiac History, %**				
Prior CAD	682 (19.1)	11 (16.4)	671 (19.1)	0.574
Prior angina	966 (27.0)	15 (22.4)	951 (27.1)	0.382
Prior Stroke	147 (4.1)	0 (0)	147 (4.1)	0.087
Congestive heart failure	141 (3.9)	0 (0)	141 (4.0)	0.094
**Other Medical History, %**				
Depression	1421 (39.8)	18 (26.9)	1403 (40.0)	0.029
Oral contraceptive use (among women)	1829 (76.3)	52 (83.9)	1777 (76.1)	0.155
Menopausal status (among women)	1225 (51.1)	18 (29.0)	1207 (51.7)	<0.001
Labor/postpartum (among women)	10 (0.4)	4 (6.5)	6 (0.3)	< .001
Alcohol use	237 (6.6)	1 (1.5)	236 (6.7)	0.087
**Clinical Presentation**				
Time to presentation > 6 hours**, %**	1495 (41.9)	25 (37.3)	1470 (41.9)	0.432
** Presenting symptom, %**				
Typical chest pain	2835 (79.4)	51 (76.1)	2789 (79.4)	0.507
Atypical chest pain	634 (17.7)	16 (23.9)	618(17.6)	0.185
**Infarct location, %**				
Anterior	1124 (31.5)	30 (44.8)	1094 (31.2)	0.018
Inferior	1309 (36.6)	13 (19.4)	1296 (37.0)	0.003
Lateral	556 (15.6)	14 (20.9)	543 (15.5)	0.225
Posterior	229 (6.4)	3 (4.5)	226 (6.4)	0.514
Right ventricle	42 (1.2)	1 (1.5)	41 (1.2)	0.808
Other	173 (4.8)	3 (3)	171 (4.9)	0.475
Type of AMI (STEMI), %	1860 (52.1)	33 (59.3)	1827 (52.1)	0.738
Peak Troponin (Median; IQR)	6.9 (1.5, 28.0)	11.8 (5.1,34.6)	6.7 (1.5, 27.8)	0.005
Initial heart rate (BPM) [Mean±SD]	83.0±20.3	78.3±18.7	83.1±20.3	0.041
Initial SBP (mm Hg) [Mean±SD]	143.7±30.9	138.6±26.7	143.7±30.9	0.126
Pre-hospital Cardiac arrest, %	205 (5.7)	4 (6.0)	201 (5.7)	0.935
Hemodynamic instability, %	309 (8.6)	4 (6.0)	305 (8.7)	0.431
Ejection fraction <40%, %	370 (10.4)	8 (11.9)	362 (10.3)	0.743
GRACE score (6-month mortality score) [Mean±SD]	74.7±18.6	71.4±17.2	74.7±18.6	0.125
Door-to-Balloon (min) (Median; IQR)	99 (60, 235)	142 (62, 1012)	99 (60, 234)	0.301
Door-to-Needle (min) (Median; IQR)	25.5 (11.0, 52.0)	37 (26.5, 138)	25 (10.5, 52)	0.409
**Hospital Interventions, %**				
Cardiac catheterization (enrolling site)	3413 (97.1)	67 (100)	3346 (97.0)	0.19
Any vessel with >50% obstruction	3097 (88.1)	46 (68.7)	3051 (88.5)	< .001
Initial TIMI flow				
PCI	2569 (73.1)	33 (49.3)	2536 (73.5)	<0.001
CABG	300 (8.4)	5 (7.5)	295 (8.4)	0.767
**Discharge Medications, %**				
Aspirin	3441 (96.3)	67 (100)	3374 (96.3)	0.235
Beta blockers	3176 (88.9)	56 (83.6)	3120 (89.0)	0.962
ACE inhibitors or ARB	2291 (64.1)	35 (52.2)	2256 (64.4)	0.014
Statin	3281 (91.9)	55 (82.1)	3230 (92.0)	0.004
**In-Hospital Complications, %**				
Re-infarction	45 (1.3)	1 (1.5)	44 (1.3)	0.863
Heart failure	243 (6.8)	2 (3.0)	241 (6.9)	0.213
Cardiac arrhythmia	65 (1.8)	1 (1.5)	64 (1.8)	0.840
Renal failure	70 (2.0)	0 (0)	70 (2.0)	0.243
Length of stay (Median days; IQR)	3 (2, 5)	3 (2, 5)	3 (2, 5)	0.817

ACE indicates angiotensin converting enzyme; ARB, Angiotensin receptor blocker; AMI acute myocardial infarction; BPM, beats per minute; BMI, body mass index; CABG, coronary artery bypass grafting; CAD, coronary artery disease; GRACE, Global Registry of Acute Coronary Events; IQR, interquartile range; PAD, peripheral artery disease; PCI, percutaneous coronary intervention; SBP, systolic blood pressure; TIA, transient ischemic attack; and TIMI, thrombolysis in myocardial infarction.

*In Spain, the default race/minority is classified as white, non-Hispanic.

### Study outcomes

We collected health status information using standardized instruments at baseline (in-hospital interview), 1-month and 12-months. Both generic (Short Form-12 [SF-12] [10], Euro-Quality of Life Scale [EQ-5D] [11]), and disease-specific (Seattle Angina Questionnaire [SAQ] [12]) health status instruments were administered to enrolled patients by trained study personnel at baseline, 1- and 12-months. The SF-12 and SAQ have 4-week recall periods, whereas the EQ-5D inquires about patients’ current health at the time of the interview.

The documentation of rehospitalizations was carried out using case-report files, which were completed by telephone interviews and chart reviews for every patient recruited; both were conducted by research nurses and project coordinators 1-year after hospitalization for AMI. Mortality events were ascertained through interviews with family members and verified with death certificates, hospital records, or obituaries.

#### SF-12 scale

This widely used instrument measures overall physical/mental health status through 12 items, which are answered along varying-length Likert scales. Both the SF-12 Physical Component Summary (PCS) and Mental Component Summary (MCS) scores were calculated for this study and range from 0 to 100, with higher scores indicating greater functioning. A score of 50 represents the US population average, with a standard deviation of 10 points. A mean difference in score of ≥5 is considered clinically significant [13–15]. The SF-12 health survey is a registered trademark of the Medical Outcomes Trust.

#### EQ-5D scale

The EQ-5D is a standardized measure of health status for clinical assessment and has been validated in AMI patients. This questionnaire has 2 parts. The first part is a descriptive section that classifies patients into 1 of 243 health states consisting of the following dimensions: mobility, self-care, usual activities, pain/discomfort, and anxiety/depression, with each dimension consisting of 3 possible levels (i.e., 1–3) representing no problems to extreme problems. These health states were then converted into an EQ-5D index score ranging from 0–100. The second part is a 20-cm Visual Analog Scale (EQ-VAS) that ranges from best imaginable state to worst state anchored at 100 and 0, respectively, with higher scores indicating better health states. The mean minimally important difference for the EQ-5D utility index score on a on a 0–100 scale is 4 (SD, 2.6) [16].

#### SAQ scale

The SAQ is a 19-item disease-specific health-related quality-of-life measure for patients with coronary artery disease [17, 18]. The 5 clinically relevant domains of the SAQ include physical limitation, angina stability, angina frequency, treatment satisfaction, and quality of life. For the purposes of this study, the physical limitation, angina frequency, treatment satisfaction, and quality-of-life domains were used. Each domain of the SAQ scores range from 0 to 100 points, with higher scores indicating higher levels of functioning, fewer symptoms, and a greater quality of life or treatment satisfaction. A change in score of 10 is interpreted as a change perceptible to patients [12]. The angina frequency score for each group was also represented as the proportion free of angina i.e angina frequency score = 100. A SAQ summary score was also calculated as the average of the physical limitation, angina frequency, and quality-of-life domains [19].

### Statistical analysis

Frequencies for categorical variables and means with standard deviations or medians with interquartile ranges for continuous variables were calculated. Statistical differences between SCAD and other AMI groups were determined with χ2 tests, t tests, and Wilcoxon rank-sum tests, as appropriate. Mean scores at baseline and 1 and 12 months were calculated and plotted between SCAD and other AMI for the SF-12, EQ-5D, and SAQ, and the changes from baseline to 12 months were represented as density plots.

To examine the difference in health status between SCAD and other AMI while taking into account the effect of time we used a longitudinal linear mixed-effects analysis fitted to each health status outcome. We included a random effect for the slope of time which accounts for the difference in time trend within a patient. Patients were organized into a multilevel longitudinal structure (patient-time) according to their health status measurements at the 3 time points (i.e., baseline and 1 and 12 months). We first examined effect of SCAD alone with the random effect for the intercept in the unadjusted model. Then in the first model (adjusted model 1) we adjusted for sociodemographic factors such as age, gender, race, marital status, education level, employment status, with the random effect for the slope of time and intercept. In the second model (adjusted model 2) we adjusted in addition for cardiovascular risk factors, comorbidities and clinical acuity (i.e. smoking status, hypertension, diabetes, dyslipidemia, body mass index ≥30 kg/m^2^, previous CAD or angina, congestive heart failure, and GRACE score). We reported the parameter estimates, 95% confidence intervals (CIs), and significance testing for the linear mixed-effects model. Forest plots were used to graphically demonstrate the effect of SCAD versus other AMI on health status through these sequential adjustment models. Covariates were missing in <3% of cases on an average. All missing data were assumed to be missing at random. Missing covariates were imputed as mean for continuous variables and mode for categorical variables.

As a sub-analysis the clinical characteristics and outcomes for SCAD versus acute myocardial infarction due to coronary artery disease (AMI-CAD) and women with SCAD vs women with other AMI were similarly calculated and reported. AMI-CAD was defined as a group likely to have an atherosclerotic nature of AMI and included patients who received revascularization (PCI or CABG) or those with >50% stenosis in a major coronary artery in the angiogram based on a previously described methodology [20].

A value of P<0.05 was considered statistically significant. All analyses were performed using R 4.0.3 (R Foundation, Vienna, Austria).

## Results and discussion

The study population consisted of 67 patients with confirmed SCAD and 3505 AMI patients with other causes. Health status data for the various instruments were available at baseline,1 and 12 months on average for 98%, 92.7%, 88.3% of SCAD patients respectively and 97.1%, 88.6%, 78.1% respectively for other AMI patients (S1 Table).

### Demographic and clinical characteristics

Table 1 compares baseline sociodemographic characteristics, cardiac risk factors, disease presentation characteristics, severity, management, discharge medications and other attributes for patients identified as SCAD versus other AMI patients.

Patients with SCAD were younger (median age (IQR) 45 (40.5–51) years vs. 48 (44–52) in other AMI, p = 0.003), more likely female (92.5% vs. 66.6% in other AMI, p<0.001), have college education or higher (73.1% vs. 51.7% in other AMI, p<0.001), work full time (71.6% vs. 50.6% in other AMI, p = 0.002) and have a household income >$100,000 (43.3% vs. 17.7% in other AMI, p<0.001). Patients with SCAD had a lower prevalence of cardiovascular risk factors including hypertension (37.3% vs 63.8% in other AMI), diabetes (7.5% vs. 35.4%), and smoking (20.9% vs. 60.5%). They were more likely premenopausal (71% vs 48.3%). Notably several SCAD cases occurred in the peripartum state (6.5% vs 0.3%) all p<0.001. There was a similar rate of ST-segment elevation myocardial infarction (STEMI) between SCAD and other AMI (59.3% and 52.1% respectively, p = 0.74). The presenting GRACE score was similar (71.4±17.2 vs 74.7±18.6 respectively, p = 0.13). Among SCAD patients 49.3% underwent PCI compared with 73.5% in other AMI (p<0.001). Angiograms were available for review in 45/67 (67.2%) SCAD cases. The left anterior descending artery was the culprit vessel in 66.7% of SCAD cases, circumflex artery in 15.6% and right coronary artery in 17.8%. There was baseline TIMI 0/1 flow in the culprit vessel in 35.5% cases. Concomitant atherosclerosis was observed in 15.5% of SCAD cases.

### Mortality and re-hospitalization

Mortality, rehospitalization and health status scores over time are shown in Table 2.

**Table 2:** Outcomes for SCAD and other AMI patients from baseline to 12-months.

	Total Population (N = 3572)	SCAD (N = 67)	Other AMI (N = 3505)	P-Value
**Mortality; N (%)**				
In-hospital mortality	4 (0.1)	0 (0)	4 (0.1)	1.000
1-month mortality	21 (0.6)	1 (1.5)	20 (0.6)	0.869
1-year mortality	72 (2.0)	1 (1.5)	71 (2.0)	1.000
**SF-12 PCS Score; Mean (SD)**				
Baseline	43.9±12.1	48.7±10.2	43.8±12.1	<0.001
1-month post-AMI	41.9 ±11.7	44.1±10.3	41.8±11.7	0.143
12-months post-AMI	44.5±12.2	50.1±9.0	44.3±12.3	<0.001
**SF-12 MCS Score; Mean (SD)**				
Baseline	45.4±12.5	49.6±12.4	45.4±12.5	0.008
1-month post-AMI	49.6±10.8	51.7±9.2	49.5±10.8	0.130
12-months post-AMI	50.2±11.0	53±10.1	50.2±11.0	0.045
**EQ-5D VAS Score; Mean (SD)**				
Baseline	64.1±21.5	69.1±21.0	64.0±21.5	0.058
1-month post-AMI	70.6±20.8	76.7±15.2	70.5±20.9	0.020
12-months post-AMI	72.5±20.8	82.2±10.2	72.3±21.0	<0.001
**EQ-5D Utility Index Score; Mean (SD)**				
Baseline	75.7±22.6	80.5±21.7	75.6±22.6	0.082
1-month post-AMI	82.4±18.2	87.8±14.2	82.3±18.3	0.017
12-months post-AMI	83.8±19.8	90.2±15.3	83.7±19.8	0.012
**SAQ Physical Limitation Score; Mean (SD)**				
Baseline	81.4±25.4	88.8±20.1	81.2±25.4	0.017
1-month post-AMI	89.8±19.5	95.8±11.8	89.7±19.6	0.015
12-months post-AMI	91.6±18.7	98.0±8.5	91.4±18.8	0.007
**SAQ Angina Frequency Score; Mean (SD)**				
Baseline	84.0±20.4	88.1±14.7	83.9±20.5	0.099
1-month post-AMI	88.9±17.8	91.3±17.5	88.8±17.8	0.278
12-months post-AMI	91.4±16.7	96.4±8.8	91.3±16.8	0.018
**SAQ Angina Frequency Score = 100; N (%)**				
Baseline	1656 (46.6)	35(47.8)	1624 (46.6)	0.849
1-month post-AMI	2027 (62.0)	42 (66.7)	1985 (61.9)	0.442
12-months post-AMI	2053 (70.4)	50 (82.0)	2003 (70.2)	0.046
**SAQ Treatment Satisfaction Score; Mean (SD)**				
Baseline	91.8±12.8	91.7±11.9	91.8±12.8	0.944
1-month post-AMI	90.6±14.3	87.7±13.3	90.6±14.3	0.109
12-months post-AMI	91.1±15.0	89.8±15.5	91.1±15.0	0.513
**SAQ Quality of Life Score; Mean (SD)**				
Baseline	56.6±24.0	60.3±22.3	56.5±24.0	0.200
1-month post-AMI	68.0±25.1	70.5±21.0	68.0±25.2	0.425
12-months post-AMI	72.4±23.1	80.7±14.7	72.2±23.2	0.005
**SAQ Summary Score; Mean (SD)**				
Baseline	73.89±18.90	79.18±15.05	73.79±18.95	0.023
1-month post-AMI	82.35±17.54	85.57±14.16	82.28±17.60	0.147
12-months post-AMI	85.48±16.03	92.25±8.06	85.34±16.13	0.001

There was no significant difference in mortality between SCAD and other AMI cohorts at 1-month (1.5% vs. 0.6%, p = 0.86) or 1-year (1.5% vs. 2%, p = 1.0) (Table 2). All cause re-hospitalization data was available for 98.5% of SCAD patients and 83.1% of other AMI patients. SCAD patients had lower 1-year all cause re-hospitalizations, but the difference was not statistically significant (21.2% in SCAD vs. 30.6% in other AMI; p = 0.10). Rehospitalization for patients with SCAD was for stable/unstable angina in 46.2%, other cardiac causes in 15.4%, and non-cardiac causes in 38.5%.

### Health status scores over time (unadjusted analyses)

**Fig 1:**
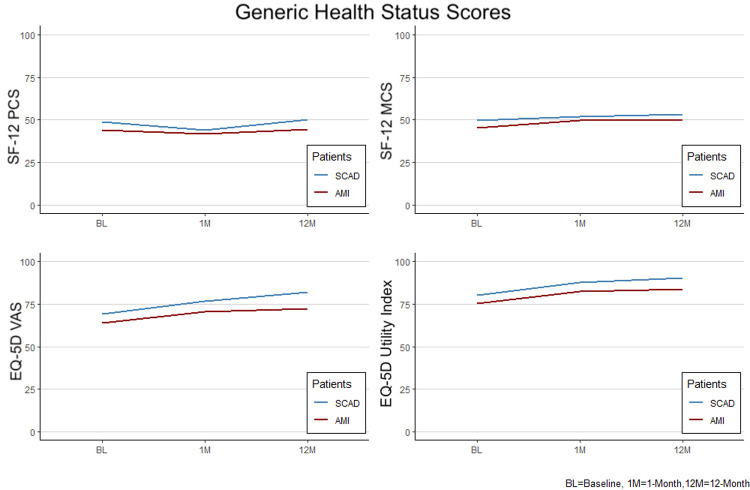
Mean health status scores from baseline to 12-months for SCAD and other AMI patients for the Short Form-12 (SF-12) and Euro-Quality of Life Scale (EQ-5D) health status measures (other AMI = red, SCAD = blue).

Generic health status: The SF-12 and EQ-5D outcomes comparing SCAD and other AMI patients are shown in Table 2 and Fig 1.

At baseline, patients with SCAD compared with other AMI had better health status scores with a higher SF-12 PCS score (48.7±10.2 vs. 43.8±12.1 for other AMI patients, p = 0.001) and a higher MCS score (49.6±12.4 vs. 45.4±12.5, p = 0.008. The SF-12 PCS and MCS scores were not statistically different at 1-month. At 12-months, SCAD patients had higher SF-12 PCS (50.1±9.0 vs. 44.3±12.3 for other AMI patients, p<0.001) and MCS score (53±10.1 vs 50.2±11.0, p = 0.045).

The EQ-5D VAS scores and EQ-5D index scores were not significantly different between SCAD an other AMI patients at baseline. At 1-month, patients with SCAD had a higher EQ-5D VAS (76.7±15.2 vs. 70.5±20.9 for other AMI patients, p = 0.02) and EQ-5D index score (87.8±14.2 vs 82.3±18.3, p = 0.02). At 12-months, SCAD patients had a higher EQ-5D VAS (82.2±10.2 vs. 72.3±21.0 for other AMI patients, p<0.001) and EQ-5D index score (90.2±15.3 vs. 83.7±19.8, p = 0.01).

**Fig 2:**
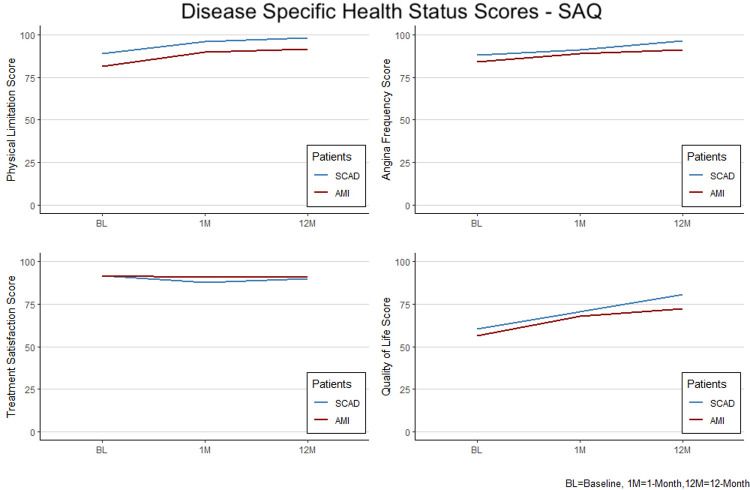
Mean health status scores from baseline to 12-months for SCAD and other AMI patients for the disease specific Seattle Angina Questionnaire (SAQ) health status measure (other AMI = red, SCAD = blue).

Disease specific health status: Disease specific SAQ health status component scores are shown in Table 2 and Fig 2.

At baseline, patients with SCAD had better physical limitation scores (88.8±20.1 vs. 81.2±25.4 for other AMI patients, p = 0.02). There was no statistical difference between the two groups in the angina frequency, proportion angina free, treatment satisfaction and quality of life scores. At 1-month, patients with SCAD had better SAQ physical limitation scores (95.8±11.8 vs. 89.7±19.6 for other AMI patients, p = 0.01). There was no statistical difference in the angina frequency, proportion angina free, treatment satisfaction, and quality of life. Lastly, at 12-months SCAD patients had better physical limitation (98.0±8.5 vs. 91.4±18.8 for other AMI patients, p = 0.007), angina frequency (96.4±8,8 vs. 91.3±16.8, p = 0.01) and quality of life scores (80.7±14.7 vs 72.2±23.2, p = 0.005), but similar treatment satisfaction scores. The proportion angina free was higher in SCAD group (82% vs 70.2%, p = 0.05).

The SAQ summary score was higher for SCAD patients at baseline (79.18±15.05 vs 73.79±18.95, p = 0.023) and at 12-months (92.25±8.06 vs 85.34±16.13, p = 0.001).

Change in Health Status Scores from Baseline to 12-Months: Density plots showing the change in health status scores from baseline to 12-months are shown in S1 and S2 Figs. The proportions of patients with improved, unchanged and worsened scores by instrument are shown in S2 Table. A similar proportion of patients experienced improvements in various health status instrument scores from baseline to 12-months; a lower proportion of SCAD patients showed improvements in SF-12 MCS scores (50% in SCAD vs. 63% in other AMI, p = 0.047) and a higher proportion showed improvements in EQ-5D VAS scores (74.6% vs. 58.9%, p = 0.016). Significant proportions of patients experienced worsening in health status scores in both SCAD and other AMI groups. Further, the mean change in health status score from baseline to 12-months was not statistically different between the two groups for most measures except the EQ-5D VAS which showed greater improvements among SCAD patients (mean difference from baseline to 12-months 14.1±20 for SCAD vs. 7.8±24.7, p = 0.02) (S3 Table).

### Effect of SCAD on health status scores (adjusted analyses)

**Fig 3:**
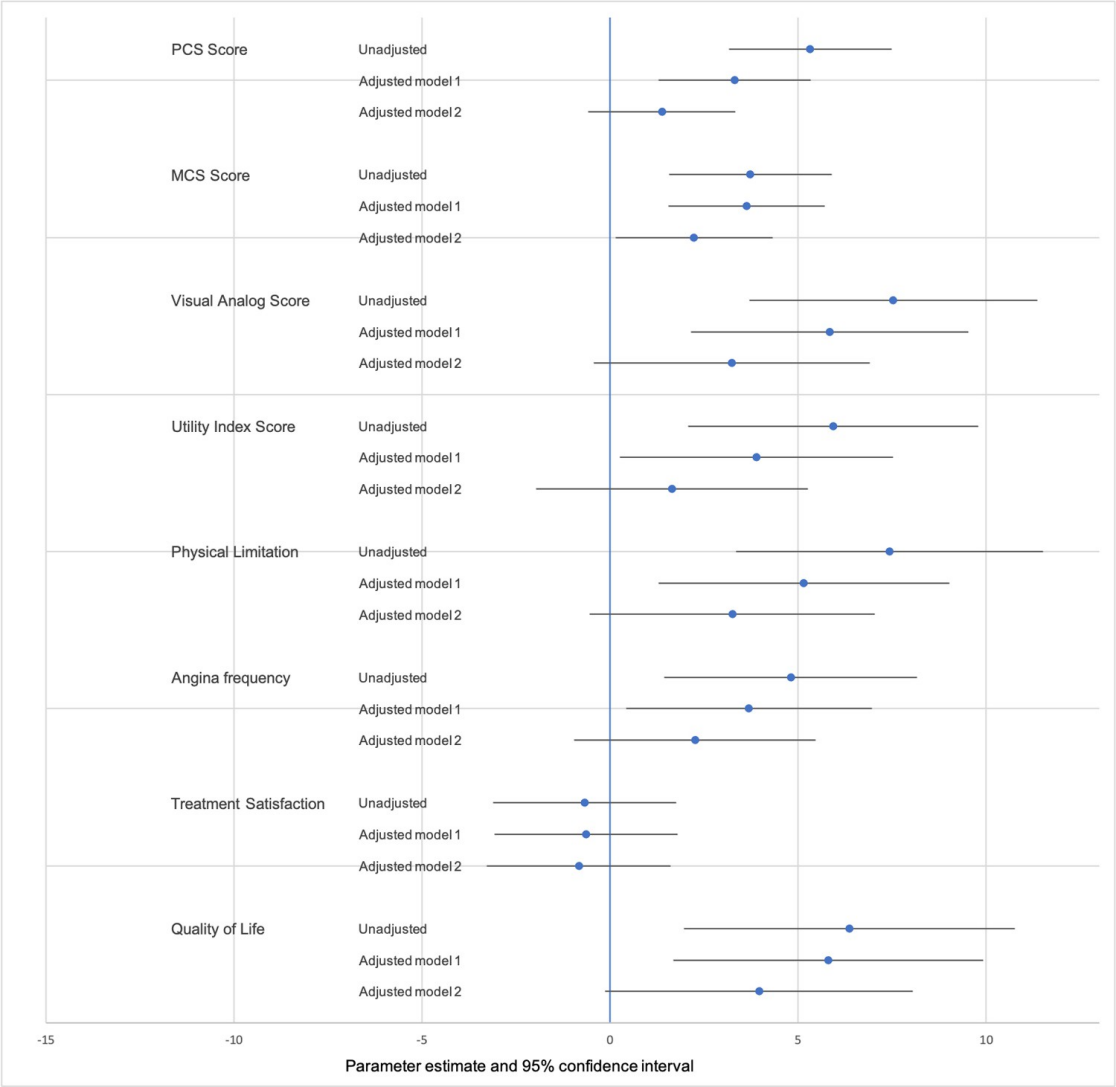
Forest plot showing the effect of SCAD versus other AMI on health status scores from baseline to 12 months. Unadjusted and with adjustment for time and other important covariates (Adjusted model 1: adjusted for sociodemographic factors; Adjusted model 2: cardiovascular risk factors, comorbidities and clinical acuity).

The results of the longitudinal linear mixed-effects model are shown in S4 Table and Fig 3.

After adjustment for sociodemographic factors SCAD patients continued to have SF-12 PCS scores that were 3.3 points higher than other AMI patients (95% CI 1.3–5.3). Similarly, scores on the SF-12 MCS, EQ-5D index score, EQ-5D VAS, SAQ-physical limitation, SAQ angina frequency, SAQ quality of life and summary score were higher for SCAD patients after adjustment for sociodemographic factors. There was no difference in treatment satisfaction scores. After further adjustment for cardiovascular risk factors, comorbidities and clinical acuity there was no difference in any of the health status outcomes between SCAD and other AMI patients except the SAQ summary score (estimate 3.4 points, 95% CI 0.4–6.4).

As a sub-analysis clinical characteristics and outcomes for SCAD versus AMI-CAD and women with SCAD vs women with other AMI are shown in S5 and S6 Tables. Of the other AMI group 88% patients were classified as AMI-CAD. The direction and magnitude of difference between these sub-group comparisons were similar to the main analysis.

Our study fills an important knowledge gap by conducting a comprehensive assessment of health status outcomes among SCAD patients and comparing to patients with other AMI. We found SCAD patients to have a lower burden of cardiovascular risk factors, and higher education, employment and income than other AMI patients, and that they present with similar acuity. Both patients with SCAD and other AMI experienced similar degrees of improvement in their health status scores over time from the initial event, however, patients with SCAD have modestly better scores than other AMI patients on most health status domains, both at presentation and at 12-months. After adjustment for socio-demographic and clinical factors, however, there were no differences in the health status scores between the two groups.

The multicenter nature of our data reduces the bias inherent to single center studies and provides more generalizable data regarding recovery after SCAD. Further, the comparison with the other AMI population provides important context to the results. The mean individual health status instrument scores for the VIRGO population was comparable to other large prospective AMI cohorts such as the Translational Research Investigating Underlying Disparities in Acute Myocardial Infarction Patients’ Health Status (TRIUMPH) registry, increasing its generalizability. For instance, the mean±SD SF-12 PCS score at baseline interview in the TRIUMPH registry was 45.7±9.9 [21], EQ-5D VAS score was 76.5±20.1, and for the SAQ components of physical limitation was 83±25, angina frequency 84±22, and quality of life 56±26 [19].

We noted that SCAD patients were younger, more likely female, premenopausal, and had lower cardiovascular risk factor burden compared with other AMI patients, as noted in other studies [22]. However, it is notable that the prevalence of risk factors in the SCAD cohort, although lower than in other AMI, is higher than that noted in other more contemporary SCAD cohorts which may point to differences in the testing patterns for SCAD between the studies [3]. The association with peripartum state was again noted [3]. Notably, SCAD patients had higher levels of education, were more likely working full time, and had higher family income. This is an important finding as socio-economic indicators such as employment and income directly affect health habits, access to quality food and housing, and access to health care, and their effect on health status and quality of life are well recognized [23]. These factors may also potentially influence recovery after the index event. Our study noted high rates of PCI in the SCAD cohort compared to more contemporary studies [5] which may be a result of VIRGO being conducted prior to the emergence of important SCAD studies raising concerns about the potential harm of angioplasty in SCAD [24]. On the other hand, the proportion of STEMI and patients with TIMI 0/1 flow was higher compared to other contemporary series which may also be contributory.

Our study shows that SCAD patients even at the time of the index event, in comparison with other AMI patients, report better physical and mental health based on the SF-12, and less physical limitation suggesting a relatively better pre-event health status among these patients. The EQ-5D scores, SAQ Angina Frequency, and SAQ Quality of Life scores, however, although higher were not significantly different. This may be expected given a lower burden of cardiovascular and other comorbidities among SCAD patients. Both SCAD and other AMI groups showed modest improvements across all measures over the 12-month period showing similar patterns of health status recovery. However, the differences in health status scores between the two groups persisted, and at 12-months, SCAD patients had better scores on nearly all generic and disease specific health status instruments than patients with other AMI. After adjustment for socio-demographic and clinical factors, however, there remained no differences in most of the health status scores between the two groups suggesting that these noted differences in health status may be partly mediated by these factors.

Overall, the results are reassuring that SCAD patients have no worse health status and in fact may fare better than other AMI patients on most health status instruments, and experience similar improvements in their health status in the 12-months following the event. Further, a better pre-event health status among SCAD patients suggests that traditional exercise and lifestyle prescriptions and cardiac rehabilitation protocols may need to be reevaluated and modified to better assist this physically active population to recover from the initial event [25, 26]. However, it is important to highlight that although many show recovery in health status scores, a significant proportion of both SCAD and AMI patients experience a worsening in health status; for instance 1 in 6 SCAD and 1 in 5 AMI patients experienced worsening in the SAQ summary score. Further study is needed to understand what patient or treatment characteristics are associated with worsening health status and develop ways to identify these patients at risk to help improve outcomes.

### Limitations

The study findings should be interpreted in the context of several potential limitations. First, performing a longitudinal study with patient interviews requires patient consent and participation. As occurs in these studies, some patients were lost to follow-up, and some patients did not respond to requests for a follow-up interview. The percentages, however, were similar for SCAD and other AMI patients (S1 Table), arguing against systematic attrition. However, it is likely that patients who were lost to follow up in both groups may have outcomes that differ than those who responded to follow up and is an important limitation. Further, patients who are not English or Spanish speaking were excluded from VIRGO and their experiences are not captured in the study. Second, as patients were required to be healthy at baseline to participate, the VIRGO cohort was unable to capture those patients who were too ill to be enrolled. Third, the VIRGO study only enrolled patients between 18–55 years of age and the findings cannot be extended to patients older than 55 years of age. However, the mean age of presentation in SCAD is between 44 and 53 years [27], and the age group included in VIRGO represents the appropriate study population for the evaluation of SCAD. Fourth, given the low number of patients in the SCAD cohort the study is susceptible to Type 2 error. Fifth, many SCAD cases may not have been identified as a CORE angiogram review for the entire AMI cohort was not performed. In addition, many SCAD cases are only apparent only on intravascular imaging and thus may not have been identified. As this study was conducted prior to several seminal articles on SCAD including a widely accepted classification scheme [28] the awareness of SCAD may have been lower reducing case identification by physicians or cases may also have misinterpreted as myocardial infarction with non-obstructive coronary arteries (MINOCA) [29]. Lastly, we only used English phrases to identify SCAD cases and thus cases in Spanish centers may not have been identified unless identified by these phrases.

## Conclusion

Patients with SCAD fare marginally better than other AMI patients on most generic and disease-specific health status instruments and have a similar health status recovery to AMI patients 12-months following the event. Baseline differences in demographic and socio-economic factors and comorbidities may explain these differences. A better pre-event health status may suggest a role of tailored interventions for these patients post SCAD event.

## Supporting information

S1 Table Availability of follow up data by instrument and time of interview. (DOCX)

S2 Table Proportions of patients with improved, unchanged and worsened scores by instrument. (DOCX)

S3 Table Mean patient level difference in scores from baseline to 12 months for SCAD and Other AMI. (DOCX)

S4 Table Longitudinal linear mixed-effects model showing the difference in health status between SCAD and other AMI unadjusted and adjusted. (Adjusted model 1: adjusted for sociodemographic factors, Adjusted model 2: sociodemographic factors, cardiovascular risk factors, comorbidities and clinical acuity).
(DOCX)

S5 Table Baseline characteristics for patients with SCAD, AMI-CAD, women with SCAD, and women with other MI. (DOCX)

S6 Table Outcomes for patients with SCAD, AMI-CAD, women with SCAD, and women with other MI from baseline to 12-months. (DOCX)

S1 Fig Distribution of change in generic health status scores from baseline to 12 months for SCAD and other AMI patients. Density plots showing the distribution of change in generic health status scores from baseline to 12 months for SCAD and other AMI patients for the Short Form-12 (SF-12) and Euro-Quality of Life Scale (EQ-5D) health status measures (AMI = red, SCAD = blue).
(DOCX)

S2 Fig Distribution of change in disease specific health status scores from baseline to 12-months for SCAD and other AMI patients. Density plots showing the distribution of change in disease specific health status scores from baseline to 12-months for SCAD and other AMI patients for the Seattle Angina Questionnaire (SAQ) health status measure (AMI = red, SCAD = blue). CVD indicates cardiovascular disease.
(DOCX)

S1 Data (XLSX)
