# Supplementary material for: Health status outcomes after spontaneous coronary artery dissection and comparison with other acute myocardial infarction: The VIRGO experience
Source: PLoS One. 2022 Mar 23;17(3):e0265624. doi: 10.1371/journal.pone.0265624 (PMC8942215; doi:10.1371/journal.pone.0265624)
Supplement: S1 Table — (DOCX) [file pone.0265624.s001.docx]

**Supplementary Table 1: Availability of follow up data by instrument and time of interview**

| Proportion (%) | Baseline | | 1-month | | 12-month | |
| --- | --- | --- | --- | --- | --- | --- |
|  | SCAD | Other AMI | SCAD | Other AMI | SCAD | Other AMI |
| Mortality | 98.5 | 98.0 | 98.5 | 97.7 | 97.0 | 96.2 |
| SF-12 PCS | 95.5 | 95.0 | 88.1 | 83.5 | 88.1 | 77.3 |
| SF-12 MCS | 98.5 | 95.9 | 88.1 | 83.5 | 88.1 | 77.3 |
| EQ-5D Visual Analog | 98.5 | 95.9 | 94.0 | 90.0 | 88.1 | 77.7 |
| EQ-5D Utility Index | 98.5 | 98.7 | 94.0 | 89.6 | 88.1 | 78.0 |
| Physical Limitation | 97.0 | 97.1 | 91.0 | 90.3 | 91.0 | 79.2 |
| Angina Frequency | 100.0 | 99.5 | 94.0 | 91.5 | 91.0 | 81.5 |
| Treatment Satisfaction | 100.0 | 99.0 | 94.0 | 90.2 | 88.1 | 78.3 |
| Quality of Life | 100.0 | 99.1 | 94.0 | 90.4 | 86.6 | 78.1 |
| SAQ summary score | 97.0 | 96.5 | 91.0 | 89.3 | 86.6 | 76.4 |
