# Supplementary material for: Health status outcomes after spontaneous coronary artery dissection and comparison with other acute myocardial infarction: The VIRGO experience
Source: PLoS One. 2022 Mar 23;17(3):e0265624. doi: 10.1371/journal.pone.0265624 (PMC8942215; doi:10.1371/journal.pone.0265624)
Supplement: S2 Table — (DOCX) [file pone.0265624.s002.docx]

**Supplementary Table 2: Proportions of patients with improved, unchanged and worsened scores by instrument**

|  |  | Improved | Unchanged | Worsened |
| --- | --- | --- | --- | --- |
| SF-12 PCS | SCAD | 53.6% | 1.8% | 44.6% |
|  | Other AMI | 50.9% | 0.7% | 48.4% |
| SF-12 MCS* | SCAD | 50.0% | 1.8% | 48.2% |
|  | Other AMI | 63.0% | 0.7% | 36.3% |
| EQ-5D VAS* | SCAD | 74.6% | 8.5% | 16.9% |
|  | Other AMI | 58.9% | 13.2% | 27.9% |
| EQ-5D Utility Index | SCAD | 51.7% | 34.5% | 13.8% |
|  | Other AMI | 50.8% | 22.6% | 26.6% |
| Physical Limitation | SCAD | 33.9% | 59.3% | 6.8% |
|  | Other AMI | 45.6% | 37.5% | 16.9% |
| Angina Frequency | SCAD | 45.9% | 47.5% | 6.6% |
|  | Other AMI | 42.0% | 40.5% | 17.6% |
| Treatment Satisfaction | SCAD | 28.8% | 40.7% | 30.5% |
|  | Other AMI | 29.4% | 41.3% | 29.3% |
| Quality of Life | SCAD | 74.1% | 10.3% | 15.5% |
|  | Other AMI | 65.9% | 11.5% | 22.6% |
| SAQ summary score | SCAD | 78.6% | 5.4% | 16.1% |
|  | AMI | 73.0% | 4.0% | 23.0% |

* P<0.05 for proportion with improved scores
