# Supplementary material for: Health status outcomes after spontaneous coronary artery dissection and comparison with other acute myocardial infarction: The VIRGO experience
Source: PLoS One. 2022 Mar 23;17(3):e0265624. doi: 10.1371/journal.pone.0265624 (PMC8942215; doi:10.1371/journal.pone.0265624)
Supplement: S3 Table — (DOCX) [file pone.0265624.s003.docx]

**Supplementary Table 3: Mean patient level difference in scores from baseline to 12 months for SCAD and Other AMI**

|  | SCAD (mean±SD) | Other AMI (mean±SD) |
| --- | --- | --- |
| SF-12 PCS | 1.59±12.64 | 0.06±12.28 |
| SF-12 MCS | 1.67±11.7 | 4.46±13.27 |
| EQ-5D VAS* | 14.08±20.01 | 7.8±24.66 |
| EQ-5D Utility Index | 10.22±20.78 | 7.25±23.4 |
| Physical Limitation | 8.4±20.69 | 9.11±26.87 |
| Angina Frequency | 8.36±15.62 | 7.15±23.52 |
| Treatment Satisfaction | -2.86±17.65 | -1.07±16.96 |
| Quality of Life | 18.68±24.4 | 15.24±27.48 |
| SAQ Summary Score | 12.04±15.71 | 10.82±20.05 |

*P<0.05
