# Supplementary material for: Health status outcomes after spontaneous coronary artery dissection and comparison with other acute myocardial infarction: The VIRGO experience
Source: PLoS One. 2022 Mar 23;17(3):e0265624. doi: 10.1371/journal.pone.0265624 (PMC8942215; doi:10.1371/journal.pone.0265624)
Supplement: S4 Table — (Adjusted model 1: adjusted for sociodemographic factors, Adjusted model 2: sociodemographic factors, cardiovascular risk factors, comorbidities and clinical acuity). (DOCX) [file pone.0265624.s004.docx]

**Supplementary Table 4: Longitudinal linear mixed-effects model showing the difference in health status between SCAD and other AMI unadjusted and adjusted** (Adjusted model 1: adjusted for sociodemographic factors, Adjusted model 2: sociodemographic factors, cardiovascular risk factors, comorbidities and clinical acuity)

| Score |  | Parameter Estimate | 95% CI Lower | 95% CI Upper | P value |
| --- | --- | --- | --- | --- | --- |
| SF-12 PCS | Unadjusted | 5.319 | 3.152 | 7.486 | <0.001 |
|  | Adjusted model 1 | 3.307 | 1.2926 | 5.327 | 0.001 |
|  | Adjusted model 2 | 1.373 | -0.5902 | 3.3366 | 0.171 |
| SF-12 MCS | Unadjusted | 3.730 | 1.5732 | 5.8871 | <0.001 |
|  | Adjusted model 1 | 3.632 | 1.5540 | 5.7099 | <0.001 |
|  | Adjusted model 2 | 2.233 | 0.1496 | 4.3159 | 0.036 |
| EQ-5D Visual Analog | Unadjusted | 7.529 | 3.703 | 11.354 | <0.001 |
|  | Adjusted model 1 | 5.837 | 2.1479 | 9.5261 | 0.002 |
|  | Adjusted model 2 | 3.239 | -0.4294 | 6.9069 | 0.084 |
| EQ-5D Utility Index | Unadjusted | 5.928 | 2.074 | 9.782 | 0.003 |
|  | Adjusted model 1 | 3.890 | 0.2541 | 7.5272 | 0.036 |
|  | Adjusted model 2 | 1.639 | -1.9770 | 5.2555 | 0.375 |
| Physical Limitation | Unadjusted | 7.423 | 3.339 | 11.506 | <0.001 |
|  | Adjusted model 1 | 5.154 | 1.2935 | 9.0169 | 0.009 |
|  | Adjusted model 2 | 3.248 | -0.5474 | 7.044 | 0.094 |
| Angina frequency | Unadjusted | 4.805 | 1.446 | 8.165 | 0.005 |
|  | Adjusted model 1 | 3.690 | 0.4220 | 6.958 | 0.027 |
|  | Adjusted model 2 | 2.253 | -0.9571 | 5.4627 | 0.169 |
| Treatment Satisfaction | Unadjusted | -0.683 | -3.120 | 1.754 | 0.583 |
|  | Adjusted model 1 | -0.635 | -3.0705 | 1.7954 | 0.609 |
|  | Adjusted model 2 | -0.834 | -3.2783 | 1.6083 | 0.504 |
| Quality of Life | Unadjusted | 6.359 | 1.958 | 10.759 | 0.005 |
|  | Adjusted model 1 | 5.800 | 1.6852 | 9.9149 | 0.006 |
|  | Adjusted model 2 | 3.962 | -0.1259 | 8.0508 | 0.058 |
| SAQ summary score | Unadjusted | 6.4487 | 3.132 | 9.766 | <0.001 |
|  | Adjusted model 1 | 5.1161 | 2.055 | 8.178 | 0.001 |
|  | Adjusted model 2 | 3.4121 | 0.425 | 6.400 | 0.025 |
