# Supplementary material for: Health status outcomes after spontaneous coronary artery dissection and comparison with other acute myocardial infarction: The VIRGO experience
Source: PLoS One. 2022 Mar 23;17(3):e0265624. doi: 10.1371/journal.pone.0265624 (PMC8942215; doi:10.1371/journal.pone.0265624)
Supplement: S5 Table — (DOCX) [file pone.0265624.s005.docx]

**Supplementary Table 5:** **Baseline characteristics for patients with SCAD, AMI-CAD, women with SCAD, and women with other MI**

|  | **SCAD (N=67)** | **AMI-CAD (N=3095)** | **P-Value** | **Women with SCAD (N=62)** | **Women with Other AMI (N=2335)** | **P-Value** |
| --- | --- | --- | --- | --- | --- | --- |
| **Socio-Demographics** |  |  |  |  |  |  |
| Age, in years (Mean±SD) | 44.5±7.1 | 47.3±6 | <0.001 | 44.5±7.3 | 47.1±6.3 | 0.001 |
| Age, in years (Median (IQR)) | 45 (40.5, 51) | 48 (44, 52) | 0.001 | 45 (40, 51) | 48 (44, 52) | 0.003 |
| **Gender, %** |  |  |  |  |  |  |
| Female | 62 ( 92.5) | 1986 ( 64.2) | <0.001 | NA | NA |  |
| **Race, %** |  |  |  |  |  |  |
| White | 53 ( 79.1) | 2463 ( 79.6) | 0.048 | 50 ( 80.6) | 1770 ( 75.8) | 0.026 |
| Black | 6 (  9.0) | 458 ( 14.8) |  | 5 (  8.1) | 435 ( 18.6) |  |
| Other | 8 ( 11.9) | 174 (  5.6) |  | 7 ( 11.3) | 130 (  5.6) |  |
| Hispanic* | 8 ( 11.9) | 216 (  7.0) | 0.185 | 6 (  9.7) | 171 (  7.3) | 0.650 |
| **Marital Status, %** |  |  |  |  |  |  |
| With partner | 46 ( 68.7) | 1805 ( 58.3) | 0.116 | 43 ( 69.4) | 1272 ( 54.5) | 0.028 |
| Without partner | 20 ( 29.8) | 1291 (41.7) |  | 19 ( 30.6) | 1063 ( 45.5) |  |
| **Education Status, %** |  |  |  |  |  |  |
| Less than high school | 4 (  6.1) | 156 (  5.1) | 0.001 | 4 (  6.6) | 134 (  5.8) | 0.001 |
| High school graduate | 13 ( 19.7) | 1288 ( 42.4) |  | 11 ( 18.0) | 959 ( 41.8) |  |
| More than high school | 49 ( 74.2) | 1594 ( 52.5) |  | 46 ( 75.4) | 1202 ( 52.4) |  |
| **Employment Status, %** |  |  |  |  |  |  |
| Working full time | 48 ( 71.6) | 1583 ( 51.1) | 0.006 | 43 ( 69.4) | 993 ( 42.5) | <0.001 |
| Working part time | 7 ( 10.4) | 340 ( 11.0) |  | 7 ( 11.3) | 305 ( 13.1) |  |
| Not working | 12 ( 17.9) | 1155 ( 37.3) |  | 12 ( 19.4) | 1026 ( 43.9) |  |
| Has health insurance, % | 61 ( 91.0) | 2466 ( 79.8) | 0.033 | 59 ( 95.2) | 1891 ( 81.1) | 0.008 |
| **Household Income** |  |  |  |  |  |  |
| <10,000 | 1 (  1.6) | 527 ( 18.5) | <0.001 | 1 (  1.7) | 465 ( 21.6) | <0.001 |
| 10,000 – 50,000 | 24 ( 38.1) | 1345 ( 47.2) |  | 21 ( 36.2) | 1061 ( 49.3) |  |
| 50,000 – 100,000 | 13 ( 20.6) | 673 ( 23.6) |  | 12 ( 20.7) | 459 ( 21.3) |  |
| >100,000 | 25 ( 39.7) | 304 ( 10.7) |  | 24 ( 41.4) | 165 (  7.7) |  |
| **Cardiac Risk Factors, %** |  |  |  |  |  |  |
| Hypertension | 25 ( 37.3) | 1993 ( 64.4) | <0.001 | 22 ( 35.5) | 1508 ( 64.6) | <0.001 |
| Diabetes | 5 (  7.5) | 1141 ( 36.9) | <0.001 | 5 (  8.1) | 924 ( 39.6) | <0.001 |
| Dyslipidemia | 40 ( 59.7) | 2094 ( 67.7) | 0.214 | 36 ( 58.1) | 1511 ( 64.7) | 0.345 |
| Smoking within last 30 days | 14 ( 20.9) | 1946 ( 62.9) | <0.001 | 12 ( 19.4) | 1424 ( 61.0) | <0.001 |
| BMI >=30kg/m2 | 18 ( 26.9) | 1544 ( 50.0) | <0.001 | 15 ( 24.2) | 1206 ( 51.8) | <0.001 |
| Family history of CAD | 35 ( 52.2) | 2263 ( 73.2) | <0.001 | 34 ( 54.8) | 1694 ( 72.6) | 0.003 |
| **Cardiac History, %** |  |  |  |  |  |  |
| Prior CAD | 11 ( 16.4) | 601 ( 19.4) | 0.646 | 10 ( 16.1) | 431 ( 18.5) | 0.763 |
| Prior angina | 15 ( 22.4) | 849 ( 27.5) | 0.433 | 14 ( 22.6) | 645 ( 27.7) | 0.460 |
| Prior Stroke | 0 (  0.0) | 121 (  3.9) | 0.184 | 0 (  0.0) | 120 (  5.1) | 0.124 |
| Congestive heart failure | 0 (  0.0) | 114 (  3.7) | 0.204 | 0 (  0.0) | 117 (  5.0) | 0.131 |
| **Other Medical History, %** |  |  |  |  |  |  |
| Depression | 18 ( 26.9) | 1224 ( 39.6) | 0.048 | 18 ( 29.0) | 1123 ( 48.1) | 0.005 |
| Oral contraceptive use (among women) | 52 ( 83.9) | 1522 ( 76.7) | 0.244 | 52 ( 83.9) | 1777 ( 76.2) | 0.209 |
| Menopausal status (among women) | 18 (29) | 1057 ( 53.2) | 0.265 | 18 ( 29.0) | 1207 ( 51.7) | 0.001 |
| Labor/postpartum (among women) | 4 (  6.0) | 4 (  0.1) | <0.001 | 4 (  6.5) | 6 (  0.3) | <0.001 |
| Alcohol use | 1 (  1.5) | 207 (  6.7) | 0.146 | 1 (  1.6) | 108 (  4.6) | 0.414 |
| **Clinical Presentation** |  |  |  |  |  |  |
| Time to presentation > 6 hours, % | 25 ( 37.3) | 1271 ( 41.2) | 0.604 | 24 ( 38.7) | 1046 ( 45.0) | 0.396 |
| Presenting symptom, % |  |  |  |  |  |  |
| Typical chest pain | 51 ( 76.1) | 2478 ( 80.1) | 0.519 | 47 ( 75.8) | 1808 ( 77.4) | 0.882 |
| Atypical chest pain | 16 ( 23.9) | 550 ( 17.8) | 0.259 | 15 ( 24.2) | 455 ( 19.5) | 0.448 |
| **Infarct location, %** |  |  |  |  |  |  |
| Anterior | 30 ( 44.8) | 1021 ( 33.0) | 0.058 | 28 ( 45.2) | 725 ( 31.0) | 0.026 |
| Inferior | 13 ( 19.4) | 1230 ( 39.7) | 0.001 | 12 ( 19.4) | 818 ( 35.0) | 0.015 |
| Lateral | 14 ( 20.9) | 510 ( 16.5) | 0.426 | 14 ( 22.6) | 369 ( 15.8) | 0.207 |
| Posterior | 3 (  4.5) | 219 (  7.1) | 0.561 | 3 (  4.8) | 131 (  5.6) | 1.000 |
| Right ventricle | 1 (  1.5) | 37 (  1.2) | 1.000 | 1 (  1.6) | 28 (  1.2) | 1.000 |
| Other | 2 (  3.0) | 131 (  4.2) | 0.845 | 2 (  3.2) | 135 (  5.8) | 0.563 |
| Type of AMI (STEMI), % | 33 ( 49.3) | 1754 ( 56.7) | 0.277 | 32 ( 51.6) | 1123 ( 48.1) | 0.676 |
| Peak Troponin  (Median; IQR) | 11.8 (5.1,34.6) | 7.8 (1.6, 32.3) | 0.0253 | 12.5 (6.3, 38.2) | 5.6 (1.3, 22.9) | <0.001 |
| Initial heart rate (BPM) [Mean±SD] | 78.3±18.7 | 82.7±19.4 | 0.067 | 78.7±19.1 | 83.9±20.4 | 0.051 |
| Initial SBP (mm Hg) [Mean±SD] | 138.6±26.70 | 143.9±31.0 | 0.160 | 136.2±24.8 | 143.0±31.9 | 0.096 |
| Pre-hospital Cardiac arrest, % | 4 (  6.0) | 183 (  5.9) | 1.000 | 3 (  4.8) | 129 (  5.5) | 1.000 |
| Hemodynamic instability, % | 4 (  6.0) | 277 (  8.9) | 0.528 | 3 (  4.8) | 209 (  9.0) | 0.369 |
| Ejection fraction <40%, % | 8 ( 11.9) | 331 ( 11.1) | 0.976 | 8 ( 12.9) | 235 ( 10.4) | 0.678 |
| GRACE score (6-month mortality score) [Mean±SD] | 71.4±17.2 | 74.0±18.3 | 0.266 | 72.5±16.9 | 75.5±18.8 | 0.224 |
| Door-to-Balloon  (Median; IQR) Units: min? | 142 (62, 1012) | 98 (59.3, 228.8) | 0.280 | 142 (66, 637) | 107.5 (63.8, 295.3) | 0.514 |
| Door-to-Needle (Median; IQR), min | 37 (26.5, 138) | 25 (10, 52) | 0.400 | 37 (26.5, 138) | 29 (10, 59) | 0.520 |
| **Hospital Interventions, %** |  |  |  |  |  |  |
| Cardiac catheterization (enrolling site) | 67 (100.0) | 3000 ( 97.3) | 0.329 | 62 (100.0) | 2222 ( 97.4) | 0.376 |
| Any vessel with >50% obstruction | 46 ( 70.8) | 3051 ( 99.1) | <0.001 | 41 ( 68.3) | 1963 ( 86.2) | <0.001 |
| PCI | 33 ( 50.0) | 2536 ( 82.1) | <0.001 | 28 ( 45.9) | 1586 ( 69.7) | <0.001 |
| CABG | 5 (  7.5) | 295 (  9.6) | 0.706 | 5 (  8.1) | 177 (  7.6) | 1.000 |
| **Discharge Medications, %** |  |  |  |  |  |  |
| Aspirin | 67 (100.0) | 3017 ( 98.8) | 0.737 | 62 (100.0) | 2230 ( 97.7) | 0.435 |
| P2Y12 inhibitor |  |  |  |  |  |  |
| Beta blockers | 56 ( 94.9) | 2829 ( 96.2) | 0.870 | 51 ( 94.4) | 2046 ( 94.1) | 1.000 |
| ACE inhibitors or ARB | 35 ( 56.5) | 2066 ( 73.2) | 0.005 | 33 ( 57.9) | 1441 ( 68.3) | 0.128 |
| Statin | 55 ( 85.9) | 2931 ( 96.6) | <0.001 | 50 ( 84.7) | 2118 ( 93.1) | 0.027 |
| **In-Hospital Complications, %** |  |  |  |  |  |  |
| Re-infarction | 1 ( 16.7) | 38 ( 21.5) | 1.000 | 1 ( 16.7) | 35 ( 24.6) | 1.000 |
| Heart failure | 2 (  3.0) | 219 (  7.1) | 0.294 | 2 (  3.3) | 180 (  7.8) | 0.288 |
| Cardiac arrhythmia | 1 (  1.5) | 59 (  1.9) | 1.000 | 1 (  1.6) | 37 (  1.6) | 1.000 |
| Renal failure | 0 (  0.0) | 56 (  1.8) | 0.520 | 0 (  0.0) | 53 (  2.3) | 0.446 |
| Length of stay (Median days; IQR) | 3 (2,5) | 3 (2,5) | 0.931 | 3 (2,5) | 3 (2, 5) | 0.982 |
