# Supplementary material for: Health status outcomes after spontaneous coronary artery dissection and comparison with other acute myocardial infarction: The VIRGO experience
Source: PLoS One. 2022 Mar 23;17(3):e0265624. doi: 10.1371/journal.pone.0265624 (PMC8942215; doi:10.1371/journal.pone.0265624)
Supplement: S6 Table — (DOCX) [file pone.0265624.s006.docx]

**Supplementary Table 6:** **Outcomes for patients with SCAD, AMI-CAD, women with SCAD, and women with other MI from baseline to 12-months**

|  | **SCAD (N=67)** | **AMI-CAD (N=3095)** | **P-Value** | **Women with SCAD (N=62)** | **Women with Other AMI (N=2335)** | **P-Value** |
| --- | --- | --- | --- | --- | --- | --- |
| **Mortality; N (%)** | | |  |  |  |  |
| In-hospital mortality | 0 (0.0) | 4 (0.1) | 1.000 | 0 (0.0) | 2 (0.1) | 1.000 |
| 1-month mortality | 1 (1.5) | 16 (0.5) | 0.818 | 1 (1.6) | 12 (0.5) | 0.778 |
| 1-year mortality | 1 (1.5) | 61 (2.0) | 1.000 | 1 (1.7) | 55 (2.5) | 1.000 |
| **SF-12 Physical Health Score; Mean (SD)** | | |  |  |  |  |
| Baseline | 48.67±10.21 | 43.68±12.01 | 0.001 | 48.28±10.48 | 42.60±12.27 | <0.001 |
| 1-month post-AMI | 44.06±10.34 | 41.78±11.68 | 0.137 | 43.48±10.58 | 40.34±11.76 | 0.052 |
| 12-months post-AMI | 50.09±9.03 | 44.37±12.21 | <0.001 | 50.32±8.70 | 42.88±12.55 | <0.001 |
| **SF-12 Mental Health Score; Mean (SD)** | | |  |  |  |  |
| Baseline | 49.57±12.36 | 45.43±12.47 | 0.009 | 49.64±12.54 | 43.84±12.75 | 0.001 |
| 1-month post-AMI | 51.70±9.15 | 49.69±10.74 | 0.154 | 51.32±9.41 | 47.94±11.18 | 0.028 |
| 12-months post-AMI | 53.05±10.11 | 50.18±10.93 | 0.046 | 52.66±10.11 | 48.91±11.43 | 0.015 |
| **EQ-5D VAS Score; Mean (SD)** | | |  |  |  |  |
| Baseline | 69.06±20.97 | 63.97±21.61 | 0.058 | 68.08±21.35 | 62.76±22.06 | 0.063 |
| 1-month post-AMI | 76.65±15.18 | 70.34±20.84 | 0.017 | 76.02±15.57 | 68.79±21.64 | 0.012 |
| 12-months post-AMI | 82.19±10.20 | 72.21±20.92 | <0.001 | 81.95±10.40 | 70.65±21.78 | <0.001 |
| **EQ-5D Utility Index Score; Mean (SD)** | | |  |  |  |  |
| Baseline | 80.46±21.71 | 75.56±22.67 | 0.082 | 79.78±22.29 | 73.02±23.32 | 0.025 |
| 1-month post-AMI | 87.85±14.21 | 82.53±18.10 | 0.021 | 87.69±14.59 | 79.83±18.96 | 0.002 |
| 12-months post-AMI | 90.23±15.31 | 83.76±19.79 | 0.013 | 89.70±15.54 | 81.37±20.28 | 0.002 |
| **SAQ Physical Limitation Score; Mean (SD)** | | |  |  |  |  |
| Baseline | 88.78±20.14 | 81.09±25.50 | 0.016 | 88.63±20.34 | 78.29±27.07 | 0.003 |
| 1-month post-AMI | 95.79±11.78 | 89.90±19.32 | 0.018 | 95.41±12.23 | 88.00±21.08 | 0.009 |
| 12-months post-AMI | 98.00±8.53 | 91.53±18.60 | 0.007 | 99.03±5.64 | 90.03±20.18 | 0.001 |
| **SAQ Angina Frequency Score; Mean (SD)** | | |  |  |  |  |
| Baseline | 88.06±14.69 | 83.61±20.57 | 0.078 | 87.74±14.87 | 82.59±21.59 | 0.062 |
| 1-month post-AMI | 91.27±17.46 | 89.06±17.66 | 0.327 | 90.52±18.01 | 87.53±18.57 | 0.226 |
| 12-months post-AMI | 96.39±8.76 | 91.23±16.90 | 0.018 | 97.37±6.69 | 90.24±17.79 | 0.003 |
| **SAQ Angina Frequency Score=100; N (%)** | | |  |  |  |  |
| Baseline | 35(47.8) | 1414 (45.9) | 0.773 | 31(53.4) | 1042 (44.8) | 0.849 |
| 1-month post-AMI | 42 (66.7) | 1791 (63.0) | 0.553 | 37 (63.8) | 1242 (58.1) | 0.195 |
| 12-months post-AMI | 50 (82.0) | 1774 (70.15) | 0.046 | 48 (84.2) | 1295 (67.6) | 0.008 |
| **SAQ Treatment Satisfaction Score; Mean (SD)** | | |  |  |  |  |
| Baseline | 91.70±11.94 | 92.24±12.09 | 0.715 | 91.83±12.11 | 91.13±13.71 | 0.688 |
| 1-month post-AMI | 87.70±13.28 | 91.10±13.77 | 0.052 | 87.18±13.52 | 89.65±15.22 | 0.220 |
| 12-months post-AMI | 89.83±15.48 | 91.45±14.47 | 0.395 | 89.84±15.84 | 90.51±15.46 | 0.751 |
| **SAQ Quality of Life Score; Mean (SD)** | | |  |  |  |  |
| Baseline | 60.32±22.29 | 56.43±24.01 | 0.189 | 60.75±22.60 | 54.56±24.68 | 0.051 |
| 1-month post-AMI | 70.50±21.00 | 68.39±24.84 | 0.503 | 69.40±20.91 | 65.22±25.92 | 0.224 |
| 12-months post-AMI | 80.75±14.66 | 71.99±23.34 | 0.005 | 80.91±15.02 | 69.87±23.61 | 0.001 |
| **SAQ Summary Score; Mean (SD)** | | |  |  |  |  |
|  | 79.18±15.05 | 73.60±19.02 | 0.019 | 79.17±15.34 | 71.71±19.89 | 0.004 |
|  | 85.57±14.16 | 82.61±17.34 | 0.186 | 84.78±14.42 | 80.36±18.42 | 0.075 |
|  | 92.25±8.06 | 85.28±16.11 | 0.001 | 92.51±8.07 | 83.74±16.83 | <0.001 |
