# Supplementary material for: Health status outcomes after spontaneous coronary artery dissection and comparison with other acute myocardial infarction: The VIRGO experience
Source: PLoS One. 2022 Mar 23;17(3):e0265624. doi: 10.1371/journal.pone.0265624 (PMC8942215; doi:10.1371/journal.pone.0265624)
Supplement: S1 Fig — Density plots showing the distribution of change in generic health status scores from baseline to 12 months for SCAD and other AMI patients for the Short Form-12 (SF-12) and Euro-Quality of Life Scale (EQ-5D) health status measures (AMI = red, SCAD = blue). (DOCX) [file pone.0265624.s007.docx]

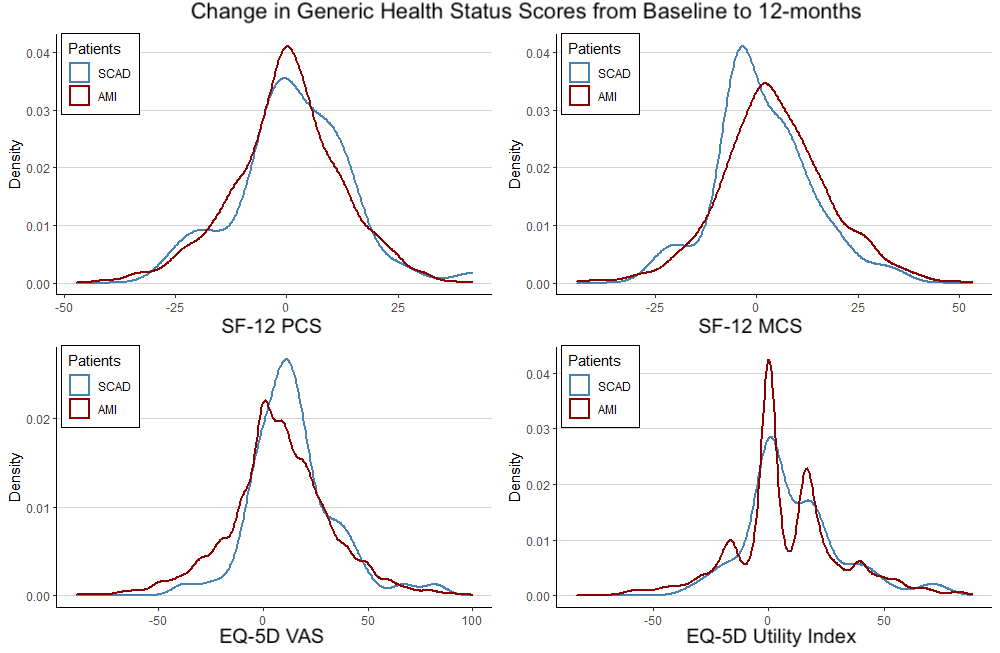


**Supplementary Figure 1**: **Distribution of change in generic health status scores from baseline to 12 months for SCAD and other AMI patients.** Density plots showing the distribution of change in generic health status scores from baseline to 12 months for SCAD and other AMI patients for the Short Form-12 (SF-12) and Euro-Quality of Life Scale (EQ-5D) health status measures (AMI=red, SCAD=blue).
