# Supplementary material for: Health status outcomes after spontaneous coronary artery dissection and comparison with other acute myocardial infarction: The VIRGO experience
Source: PLoS One. 2022 Mar 23;17(3):e0265624. doi: 10.1371/journal.pone.0265624 (PMC8942215; doi:10.1371/journal.pone.0265624)
Supplement: S2 Fig — Density plots showing the distribution of change in disease specific health status scores from baseline to 12-months for SCAD and other AMI patients for the Seattle Angina Questionnaire (SAQ) health status measure (AMI = red, SCAD = blue). CVD indicates cardiovascular disease. (DOCX) [file pone.0265624.s008.docx]

**
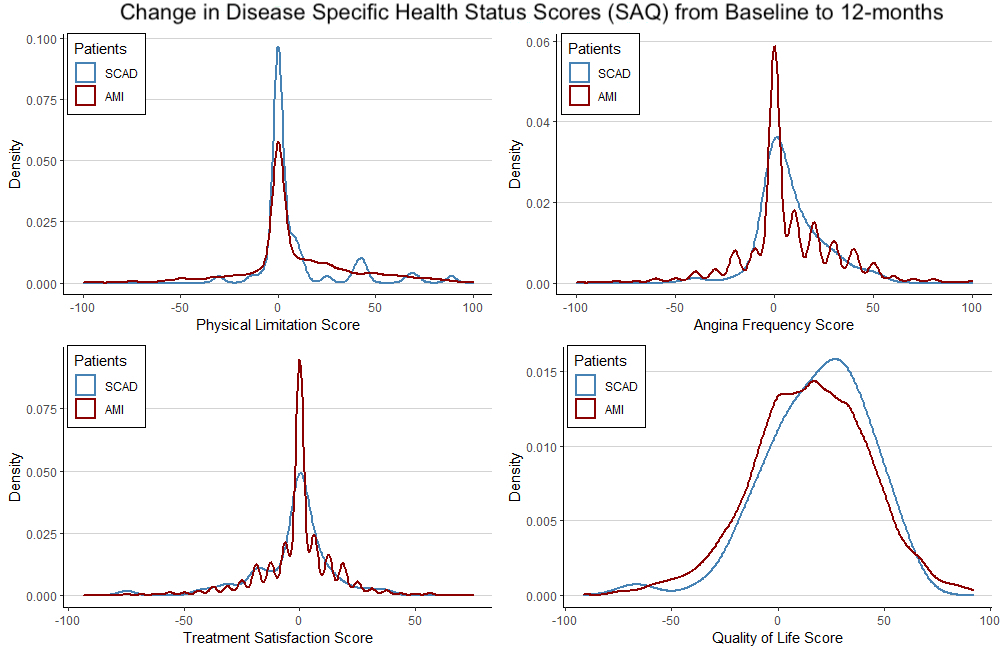
**

**Supplementary Figure 2**. **Distribution of change in disease specific health status scores from baseline to 12-months for SCAD and other AMI patients.** Density plots showing the distribution of change in disease specific health status scores from baseline to 12-months for SCAD and other AMI patients for the Seattle Angina Questionnaire (SAQ) health status measure (AMI=red, SCAD=blue). CVD indicates cardiovascular disease.
